# Supplementary material for: A somatic proteoglycan controls Notch-directed germ cell fate
Source: Nat Commun. 2021 Nov 18;12:6708. doi: 10.1038/s41467-021-27039-4 (PMC8602670; doi:10.1038/s41467-021-27039-4)
Supplement: Supplementary file 1 — Supplementary Information [file 41467_2021_27039_MOESM1_ESM.pdf]

Supplementary Materials for

A Somatic Proteoglycan Controls Notch-Directed Germ Cell Fate

Sandeep Gopal*, Aqilah Amran, Andre Elton, Leelee Ng and Roger Pocock*

Correspondence to: roger.pocock@monash.edu and sandeep.gopal@monash.edu

This PDF file includes:

Figures. S1 to S10
Table S1

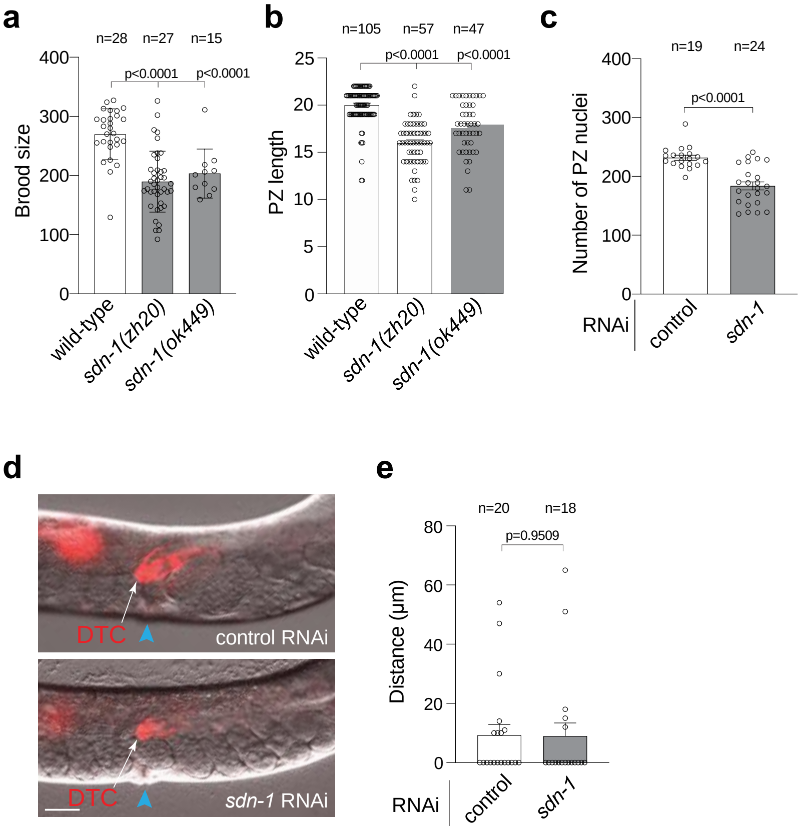

Fig. S1. ***sdn-1*** **loss-of-function germline analysis**

(A) Brood size of wild-type, *sdn-1(zh20)* and *sdn-1(ok449)* hermaphrodites. Data are expressed as mean ± s.e.m. and statistical significance was assessed by ordinary one-way ANOVA.

(B) PZ length of wild-type, *sdn-1(zh20)* and *sdn-1(ok449)* adult hermaphrodites. Data are expressed as mean ± s.e.m. and statistical significance was assessed by ordinary one-way ANOVA.

(C) 3D germline quantification of PZ nuclei in adult hermaphrodite (+/- *sdn-1* RNAi). Data are expressed as mean ± s.e.m. and statistical significance was assessed by two-tailed unpaired t-test.

(D-E) Immunofluorescence images (D) and quantification (E) distal tip cell position in adult hermaphrodites (+/- *sdn-1* RNAi) with respect to vulva (blue arrowhead). Data are expressed as mean ± s.e.m. and statistical significance was assessed by two-tailed unpaired t-test. For all panels, n refers to number of animals analyzed. P-values as indicated.

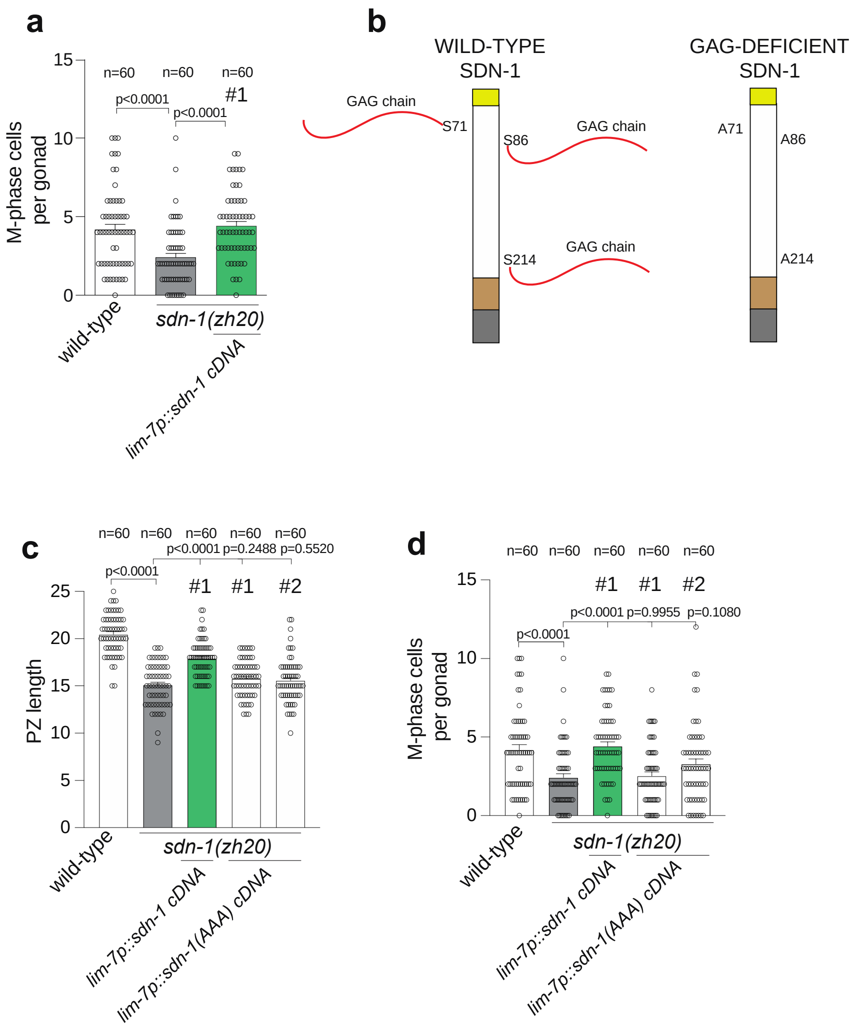

Fig. S2. *sdn-1* mutant rescue experiments

(A) Quantification of M-phase cells in distal germlines of wild-type, *sdn-1(zh20)* and *sdn-1(zh20); lim-7p::sdn-1 cDNA* adult hermaphrodites. The *lim-7p::sdn-1 cDNA* rescue line corresponds to the same line in Figure 1F. Data are expressed as mean ± s.e.m. and statistical significance was assessed by ordinary one-way ANOVA.

(B) Schematic of wild-type SDN-1 and GAG-deficient SDN-1. Attachment sites (S71, S86 and S214) are shown with GAG chains as red lines in wild-type. Alanine mutations that prevent GAG attachment are shown in the GAG-deficient SDN-1. Yellow - signal sequence; White - ectodomain; Brown - transmembrane domain; Grey - cytoplasmic domain.

(C) Quantification of PZ length in distal germlines of adult hermaphrodites in wild-type, *sdn-1(zh20)* and *sdn-1(zh20); lim-7p* rescue lines expressing wild-type SDN-1 or GAG-chain deficient SDN-1(AAA). Independent rescue lines represented by # (#1 line for wild type SDN-1 corresponds to the same line in Figure 1F). Data are expressed as mean ± s.e.m. and statistical significance was assessed by ordinary one-way ANOVA.

(D) Quantification of M-phase cells in distal germlines of adult hermaphrodites in wild-type, *sdn-1(zh20)* and *sdn-1(zh20); lim-7p* rescue lines expressing wild-type SDN-1 or GAG-chain deficient SDN-1(AAA). Independent rescue lines represented by # (#1 line for wild type SDN-1 corresponds to the same line in Figure 1F). Data are expressed as mean ± s.e.m. and statistical significance was assessed by ordinary one-way ANOVA. For all panels, n refers to number of animals analyzed. P-values as indicated.

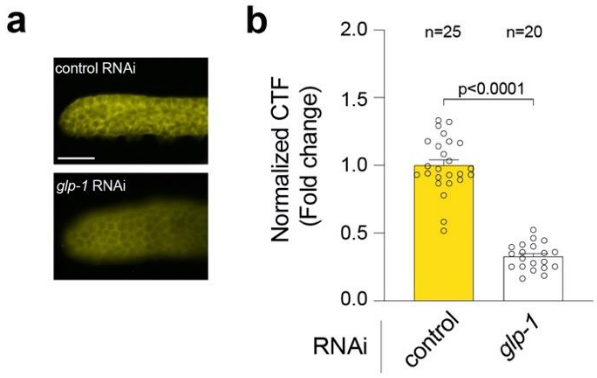

Fig. S3. Validation of *glp-1* RNAi

(A-B) Immunofluorescence images (A) and quantification (B) of GLP-1::V5 expression in the PZ of wild-type (+/- *glp-1* RNAi). Data are expressed as mean ± s.e.m. and statistical significance was assessed by two-tailed unpaired t-test. Scale bar 20µm. n refers to number of animals analyzed. P-values as indicated.

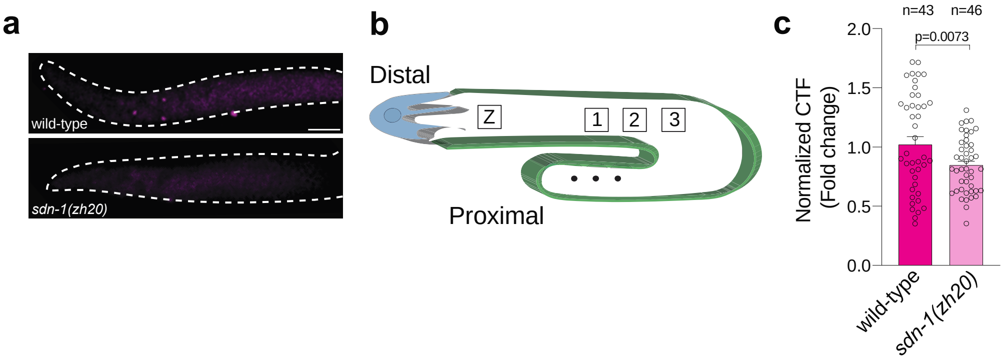

Fig. S4. *glp-1* transcript analysis by smFISH

(A-C) Immunofluorescence images (A), schematic of image analysis (B) and quantification (C) of *glp-1* smFISH expression in the PZ of wild-type and *sdn-1(zh20)* adult hermaphrodites. *glp-1* transcripts = magenta (A). smFISH signal was quantified from three areas (boxes 1-3) while considering an area in the distal end as the background (box Z) (B). Data are expressed as mean ± s.e.m. and statistical significance was assessed by two-tailed unpaired t-test. Scale bar = 20µm. n refers to number of animals analyzed. P-value as indicated.

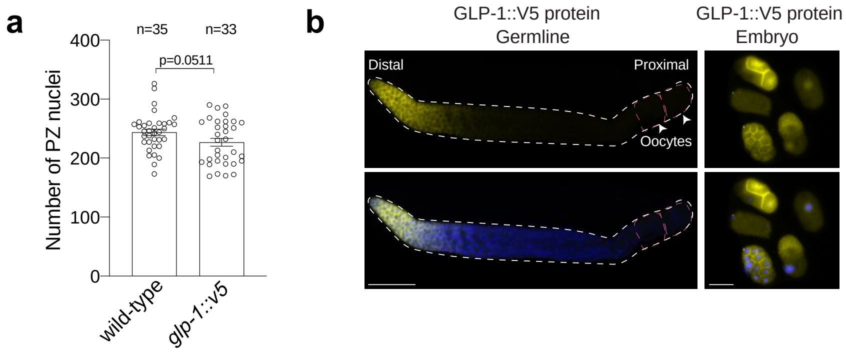

Fig. S5. Analysis of CRISPR-Cas9 generated GLP-1::V5 strain

(A) Quantification of PZ nuclei of wild-type and CRISPR-Cas9-tagged *glp-1::v5* adult hermaphrodites. Data are expressed as mean ± s.e.m. and statistical significance was assessed by two-tailed unpaired t-test. P-value as indicated. n refers to number of animals analyzed.

(B) Images of GLP-1::V5 in adult germlines and embryos. 30 germlines and 20 embryos were analysed. Performed in duplicate. Scale bars = 25µm.

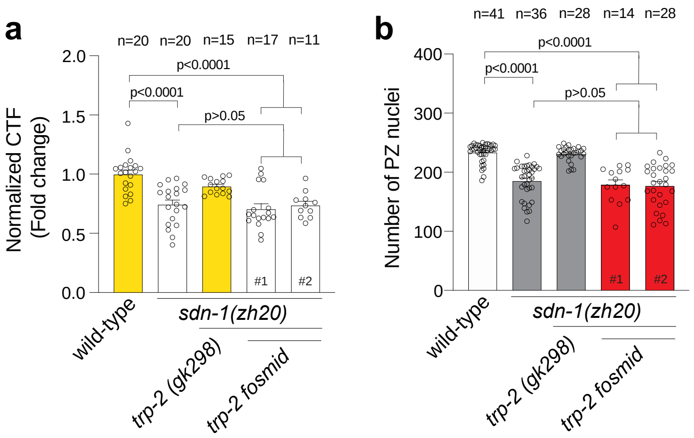

Fig. S6. *trp-2* fosmid rescue analysis

(A) Quantification of GLP-1::V5 protein in wild-type, *sdn-1(zh20)* and *sdn-1(zh20); trp-2(gk298)* (+/- *trp-2* fosmid) in adult hermaphrodite germlines. Data are expressed as mean ± s.e.m. and statistical significance was assessed by ordinary one-way ANOVA.

(B) Quantification of PZ nuclei in wild-type*, sdn-1(zh20) and sdn-1(zh20); trp-2(gk298)* (+/- *trp-2* fosmid) in adult hermaphrodite germlines. Data are expressed as mean ± s.e.m. and statistical significance was assessed by ordinary one-way ANOVA. n refers to number of animals analyzed. P-values as indicated.

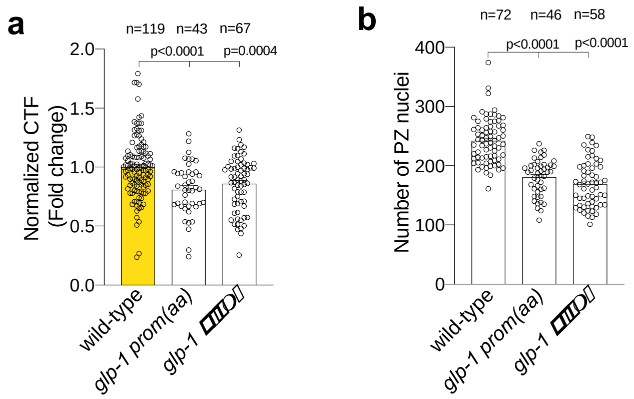

Fig. S7. *glp-1* promoter analysis

(A) Quantification of GLP-1::V5 protein in wild-type, *glp-1prom(aa)* and *glp-1prom*∆ adult hermaphrodite germlines. Data are expressed as mean ± s.e.m. and statistical significance was assessed by ordinary one-way ANOVA.

(B) Quantification of PZ nuclei in wild-type, *glp-1prom(aa)* and *glp-1prom*∆ adult hermaphrodites. Data are expressed as mean ± s.e.m. and statistical significance was assessed by ordinary one-way ANOVA. n refers to number of animals analyzed. P-values as indicated.

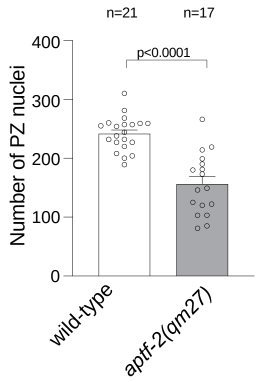

Fig. S8. PZ nuclei analysis of *aptf-2(qm27)* animals

Quantification of PZ nuclei in wild-type and *aptf-2(qm27)* adult hermaphrodites. Data are expressed as mean ± s.e.m. and statistical significance was assessed by two-tailed unpaired t-test. n refers to number of animals analyzed. P-value as indicated.

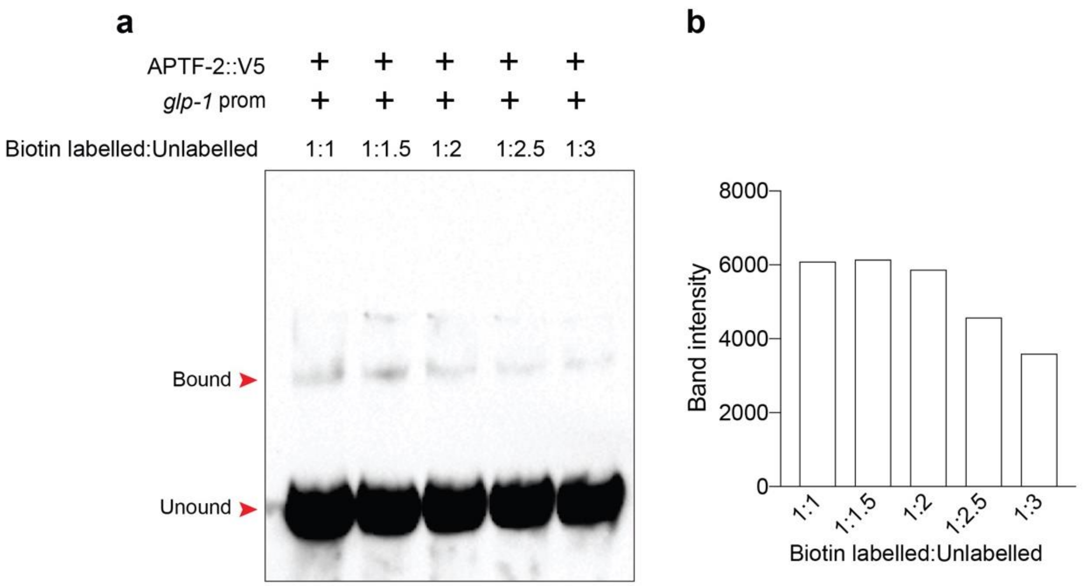

Fig. S9. *glp-1* EMSA analysis

(A-B) EMSA competition experiments. APTF-2::V5 protein produced in HEK293T cells and the TFAP2 consensus motif in the *glp*-1 promoter (A). DNA bound and unbound with APTF-2::V5 is marked. Biotin-labelled wild-type *glp-1* promoter is competed away with increasing amount of unlabeled *glp-1* promoter. Quantification of shifted *glp-1* promoter with increasing amount of unlabeled *glp-1* promoter (B).

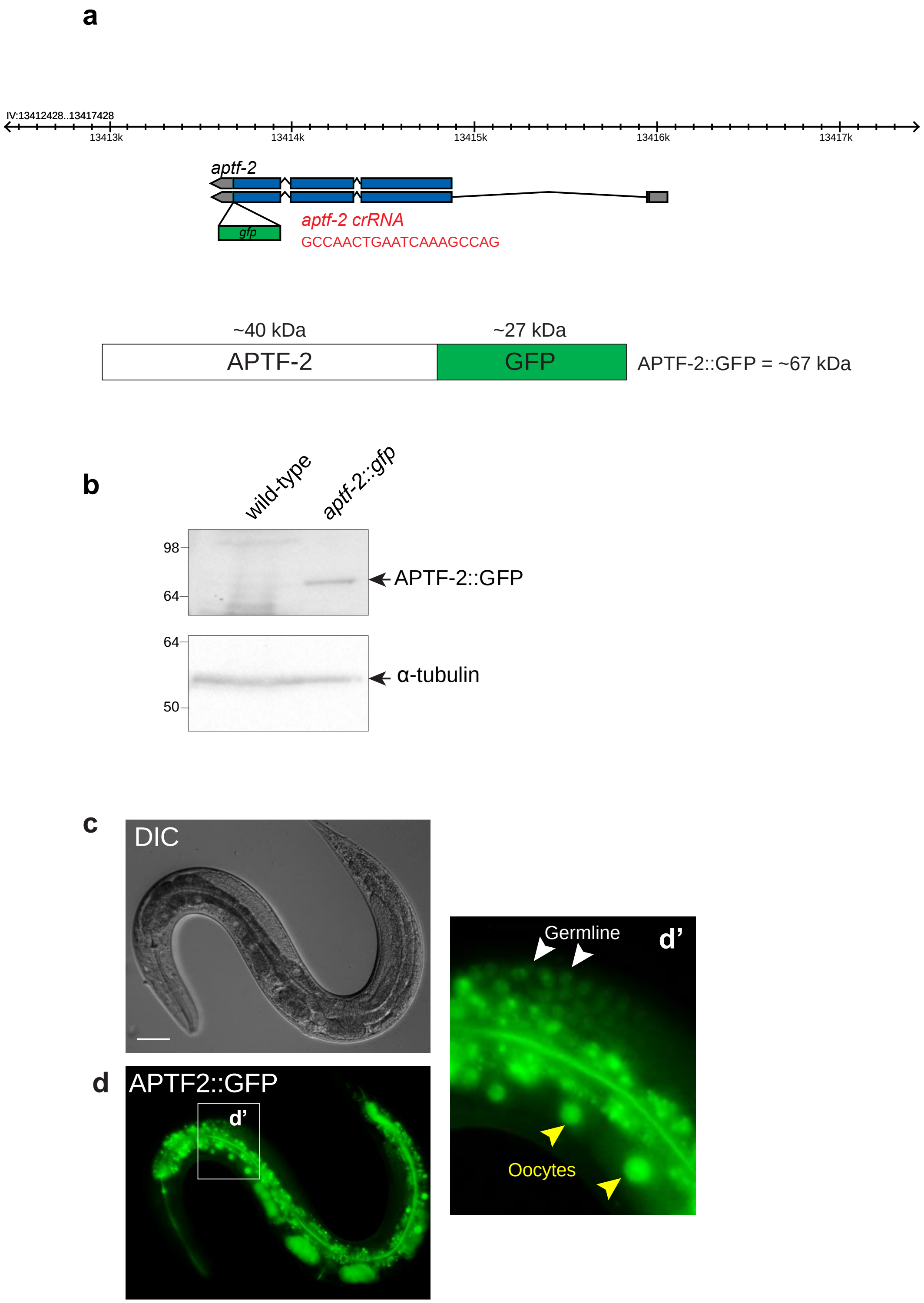

Fig. S10. APTF-2::GFP expression analysis

(A) *aptf-2* genomic locus (upper image) showing 3'-end tagging of the gene (crRNA used for Cas9 cleavage in red). Schematic of APTF-2 protein (lower image) showing

approximate molecular weight of the fusion protein following CRISPR/Cas9 insertion of GFP.

(B) Western blot of endogenously tagged APTF-2::GFP from mixed-stage worms. APTF-2::GFP protein is the correct size (APTF-2 = ~40kDa, GFP = 27kDa, combined = ~67kDa). Top blot - anti-GFP antibody; bottom blot - anti-tubulin loading control. Performed in duplicate.

(C-D) Endogenous APTF-2::GFP expression in the adult hermaphrodite germline. DIC image of a young adult hermaphrodite (C), fluorescence image of the same worm (D) and zoomed image of the boxed region (D’) showing expression in the germline (white arrowheads) and oocytes (yellow arrowheads). Scale bar = 50µm.

Table S1. Materials used.

**Reagents**	**Company**	**Catalogue number**
Formaldehyde (37%)	Sigma	F8775
Lipofectamine 2000	Thermo Fisher	11668019
UltraPure™ Agarose	Thermo Scientific	16500-500
Ampicillin sodium salt	Sigma	A9518
DMEM	Life Technologies	11960044
L-glutamine	Life Technologies	25030-081
Fluoroshield	Sigma Aldrich	F6182
Formamide, Deionized	Merck Millipore	S4117
DAPI readymade solution	Sigma Aldrich	MBD0015
Alexa Fluor™ Plus 555 Phalloidin	Thermofisher	A30106
Goat serum (10mL)	Sigma Aldrich	G9023
Epstein-Barr Nuclear Antigen (EBNA)	Thermofisher	Part of 20148
**Antibody**	**Company**	**Catalogue number**
Mouse monoclonal anti-V5 (clone SV5-pk1) (1:1000)	BioRad	MCA1630
Mouse monoclonal anti-α-tubulin (clone 12G10) (1:200)	Developmental Studies Hybridoma Bank	AB1157911
Mouse-polyclonal anti-GFP (1:1000)	Sigma Aldrich	11814460001
Rabbit-monoclonal anti-pH3 (1:1000)	Sigma Aldrich	06570
Goat anti-Mouse IgG (H+L) Secondary Antibody, HRP conjugate (1:1000)	Life Technologies	32430
**Bacterial strain**	**Source**	**Identifier**
Bacteria *Escherichia coli* HT115	CAENORHABDITIS GENETICS CENTER (CGC)	N/A
Bacteria *Escherichia coli* OP50	CGC	N/A
***C. elegans* strain**	**Source**	**Identifier**
sdn-1(zh20)	CGC	AH205
trp-2(gk298)	CGC	*VC602*
glp-1::v5(q1000)	Kimble lab	JK5933
sdn-1(ok449)	CGC	RB690
sdn-1(zh20); *trp-2(gk298) glp-1::v5(q1000)*	This study	RJP3846
sdn-1(zh20); rpIs121(lim-7p::sdn-1::gfp::tbb-2) line 1	This study	RJP3867
sdn-1(zh20); rpIs121(lim-7p::sdn-1::gfp::tbb-2) line 2	This study	RJP3866
sdn-1(zh20); rpIs154(mex-5p::sdn-1::gfp::tbb-2) line 1	This study	RJP3857
sdn-1(zh20); rpIs154(mex-5p::sdn-1::gfp::tbb-2) line 2	This study	RJP3855

sdn-1(zh20) rpIs114(lag-2p::sdn-1::gfp::tbb-2) line 1	This study	RJP3863
sdn-1(zh20); rpIs114(lag-2p::sdn-1::gfp::tbb-2) line 2	This study	RJP3858
sdn1(zh20); rpIs157(lim-7p::sdn-1SA::gfp::tbb-2) line 1	This study	RJP3746
sdn1(zh20); rpIs157(lim-7p::sdn-1SA::gfp::tbb-2) line 2	This study	RJP3747
sdn-1(zh20); *glp-1::v5(q1000)*	This study	RJP3845
trp-2(gk298) glp-1::v5(q1000)	This study	RJP4118
glp-1::v5(q1000)promΔ	This study	*RJP4729*
sdn1(zh20); glp-1::V5(q1000)promΔ	This study	RJP4730
glp-1::v5(q1000)prom(aa)	This study	*RJP4744*
glp1::v5(q1000); aptf-2:: gfp	This study	RJP4035
sdn-1(zh20); glp-1::V5(q1000); aptf-2::gfp	This study	RJP4731
glp-1::v5(q1000); rpEx2183(sdn-1p::gfp)	This study	RJP5186
glp-1::v5(q1000); rpEx2184(lim-7p::gfp)	This study	RJP5187
sdn-1(zh20); *glp-1::v5(q1000); rpEx2184(lim-7p::gfp)*	This study	RJP5188
trp-2p::mCherry (lskEx383)	PMID: 29883446	KHK667
sdn-1(zh20); *trp-2(gk298) glp-1::v5(q1000); rpEx2185(lim-7p::trp-2)* line 1	This study	RJP5189
sdn-1(zh20); *trp-2(gk298) glp-1::v5(q1000); rpEx2186(lim-7p::trp-2)* line 2	This study	RJP5190
qIs154 [lag-2p::MYR::tdTomato + ttx-3p::GFP] V	PMID: 24586318	JK4472
**Mammalian Cell Lines**	**Source**	**Identifier**
HEK293T	N/A	N/A
Wild-type mouse embryonic fibroblast (MEF)	N/A	N/A
Syndecan-4 knockout MEF	N/A	N/A
**Commercial Kit**	**Company**	**Catalogue number**
LightShift™ Chemiluminescent EMSA Kit	Thermofisher	20148
Pierce™ Biotin 3' End DNA Labelling Kit	Thermofisher	89818
NE-PER™ Nuclear and Cytoplasmic Extraction Reagents	Thermofisher	78833
**Plasmid**	**Source**	**Identifier**
pCDNA3.2-aptf-2-v5	This study	N/A
minimos-mex-5p::sdn-1::tbb-2-UTR	Bulow lab	N/A
minimos-lag-2p::sdn-1::tbb-2-UTR	Bulow lab	N/A
minimos-lim-7p::sdn-1::tbb-2-UTR	Bulow lab	N/A
rab-3p::mCherry::unc-54UTR	This study	N/A
myo-2p::mCherry::unc-54UTR	This study	N/A
myo-3p::mCherry::unc-54UTR	This study	N/A

eft-3p::mos1transposase::tbb-2UTR	This study	N/A
hsp16.41p::peel-1::tbb-2UTR	This study	N/A
sdn-1p::gfp	This study	N/A
lim-7p::gfp	This study	N/A
lim-7p::trp-2 cDNA	This study	N/A
**Oligonucleotide**	**Source**	**Identifier**
glp-1-prom F<br>5'aacacactcagaaaatgggcggagtatgccacccggccttcattcgtcgctccttgtctt3'	This study	oSG168
glp-1-prom R<br>5'aagacaaggagcgacgaatgaaggccgggtggcatactccgcccattttctgagtgtgtt3'	This study	oSG169
glp-1-promΔ F<br>5'aacacactcagaaaatgggcggagtatgcggccttcattcgtcgctccttgtctt3'	This study	oSG170
glp-1-promΔ R<br>5'aagacaaggagcgacgaatgaaggccgcatactccgcccattttctgagtgtgtt3'	This study	oSG171
Control DNA duplex<br>5'tagcatatgcta3'<br>3'atcgtatacgat5'	Thermofisher	Part of 20148
crRNA for aptf-2-gfp<br>gccaactgaatcaaagccag	This study	N/A
crRNA for *glp-1* mutations<br>aatgggcggagtatgccacc	This study	N/A
sdn-1 promoter F<br>5'gaaatgaaataagctttcacctgatagcgcgtaacg3'	This study	N/A
sdn-1 promoter R<br>5'ccaatcccggggatccattgtcttgttggtgtaatcacc3'	This study	N/A
lim-7p::gfp reporter F<br>5'cgactctagaggatcagtacttgtgccttgattctcctg3'	This study	N/A
lim-7p::gfp reporter R<br>5'tcattttttctaccgccaatcgacagccggcac3'	This study	N/A
lim-7p::trp-2 F<br>5'aggacccttggctagcatgacatcatcagaagcatccgag3'	This study	N/A

lim-7p::trp-2 R 5’gatatcaataccatggtcataaaagttcatcagtgtcgtc3’	This study	N/A
**Software**	**Source**	**Identifier**
ImageJ 1.47n	National Institute of Health, USA	N/A
Imaris 9.1.2	Bitplane	N/A

Uncropped blots

All blots were detected by biotin labelling of oligos and chemiluminescent EMSA kit as explained in supplementary materials.

Figure S9A

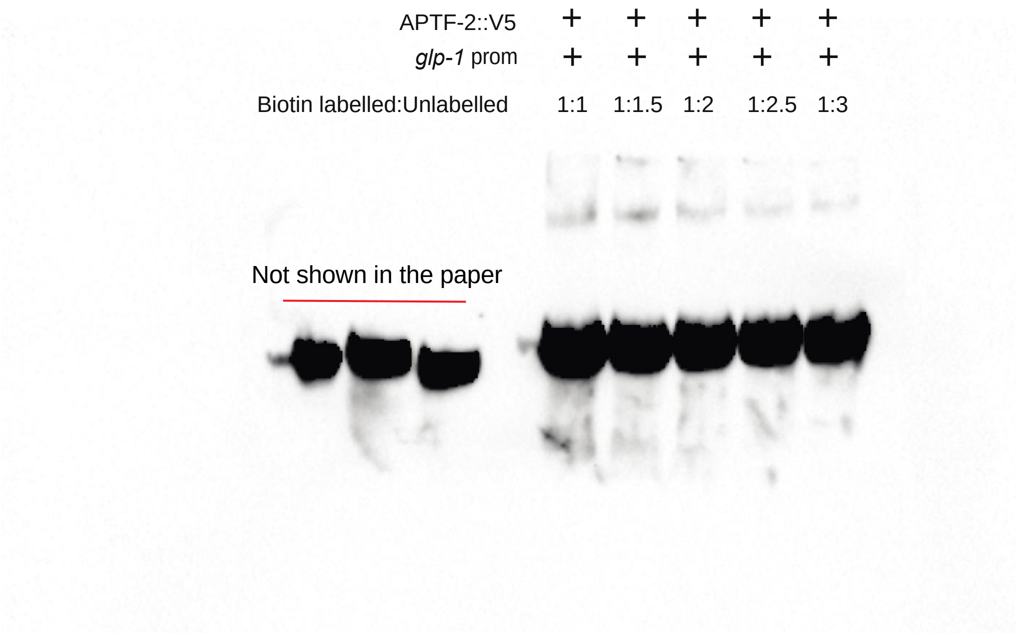

Uncropped images of Western blots detecting APTF-2::GFP

Supplementary Figure 10B - Top panel high exposure

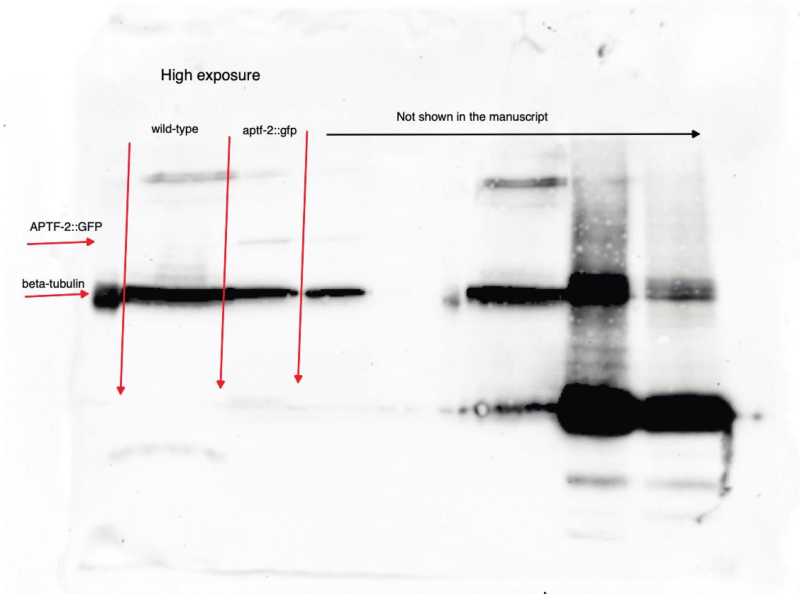

Supplementary Figure 10B - Bottom panel low exposure

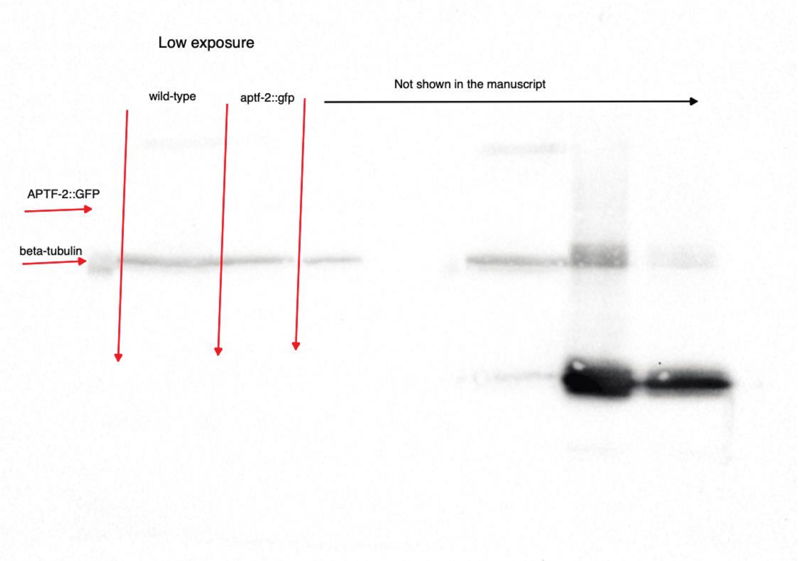

253
