# Supplementary material for: A somatic proteoglycan controls Notch-directed germ cell fate
Source: Nat Commun. 2021 Nov 18;12:6708. doi: 10.1038/s41467-021-27039-4 (PMC8602670; doi:10.1038/s41467-021-27039-4)
Supplement: Supplementary file 3 — Reporting Summary [file 41467_2021_27039_MOESM3_ESM.pdf]

nature portfolio

Corresponding author(s): Roger Pocock

Last updated by author(s): Jun 27, 2021

Reporting Summary

Nature Portfolio wishes to improve the reproducibility of the work that we publish. This form provides structure for consistency and transparency in reporting. For further information on Nature Portfolio policies, see our Editorial Policies and the Editorial Policy Checklist.

Statistics

For all statistical analyses, confirm that the following items are present in the figure legend, table legend, main text, or Methods section.

n/a	Confirmed	
☐	☒	The exact sample size (*n*) for each experimental group/condition, given as a discrete number and unit of measurement
☒	☐	A statement on whether measurements were taken from distinct samples or whether the same sample was measured repeatedly
☐	☒	The statistical test(s) used AND whether they are one- or two-sided<br>*Only common tests should be described solely by name; describe more complex techniques in the Methods section.*
☒	☐	A description of all covariates tested
☐	☒	A description of any assumptions or corrections, such as tests of normality and adjustment for multiple comparisons
☐	☒	A full description of the statistical parameters including central tendency (e.g. means) or other basic estimates (e.g. regression coefficient) AND variation (e.g. standard deviation) or associated estimates of uncertainty (e.g. confidence intervals)
☐	☒	For null hypothesis testing, the test statistic (e.g. *F*, *t*, *r*) with confidence intervals, effect sizes, degrees of freedom and *P* value noted<br>*Give P values as exact values whenever suitable.*
☒	☐	For Bayesian analysis, information on the choice of priors and Markov chain Monte Carlo settings
☒	☐	For hierarchical and complex designs, identification of the appropriate level for tests and full reporting of outcomes
☒	☐	Estimates of effect sizes (e.g. Cohen's *d*, Pearson's *r*), indicating how they were calculated

Our web collection on statistics for biologists contains articles on many of the points above.

Software and code

Policy information about availability of computer code

Data collection	Zen (Zeiss version 2.0.0.0 Blue) and Imaris 9.1.2 software were used to acquire images.
Data analysis	Statistical analysis was conducted using Graph Pad (Prism). Imaris 9.1.2 and ImageJ 1.47n software were used for image analysis.

For manuscripts utilizing custom algorithms or software that are central to the research but not yet described in published literature, software must be made available to editors and reviewers. We strongly encourage code deposition in a community repository (e.g. GitHub). See the Nature Portfolio guidelines for submitting code & software for further information.

Data

Policy information about availability of data

All manuscripts must include a data availability statement. This statement should provide the following information, where applicable:

- Accession codes, unique identifiers, or web links for publicly available datasets
- A description of any restrictions on data availability
- For clinical datasets or third party data, please ensure that the statement adheres to our policy

All data is available in the main text or the supplementary materials. In addition, Source Data are provided. There are no accession codes, unique identifiers or weblinks in our study and no restrictions on data availability.

Field-specific reporting

Please select the one below that is the best fit for your research. If you are not sure, read the appropriate sections before making your selection.

☒ Life sciences ☐ Behavioural & social sciences ☐ Ecological, evolutionary & environmental sciences

For a reference copy of the document with all sections, see nature.com/documents/nr-reporting-summary-flat.pdf

Life sciences study design

All studies must disclose on these points even when the disclosure is negative.

Sample size	We determined the same size based on similar experimental setups described in previous publication Dev Biol. 2017 Dec 15;432(2):222-228. Based on this study, the same size used in the current study is sufficient to assess statistical significance.
Data exclusions	No data were excluded from this study.
Replication	Multiple trials/biological of each experiment were conducted. Replicates are indicated in the figure legends or where combined for data representation.
Randomization	Samples were randomly chosen and allocated into experiments groups. For C. elegans experiments, animals from each strain or transgenic line were randomly selected.
Blinding	Experiments were conducted by multiple investigators blinded to genotype.

Reporting for specific materials, systems and methods

We require information from authors about some types of materials, experimental systems and methods used in many studies. Here, indicate whether each material, system or method listed is relevant to your study. If you are not sure if a list item applies to your research, read the appropriate section before selecting a response.

Materials & experimental systems

n/a	Involved in the study	
☐	☒	Antibodies
☐	☒	Eukaryotic cell lines
☒	☐	Palaeontology and archaeology
☐	☒	Animals and other organisms
☒	☐	Human research participants
☒	☐	Clinical data
☒	☐	Dual use research of concern

Methods

n/a	Involved in the study	
☒	☐	ChIP-seq
☒	☐	Flow cytometry
☒	☐	MRI-based neuroimaging

Antibodies

Antibodies used	Mouse monoclonal anti-V5 (clone SV5-pk1) BioRad MCA1630 - 1:1000<br>Mouse monoclonal anti-alpha-tubulin (clone 12G10) Developmental Studies Hybridoma Bank AB1157911 - 1:200<br>Mouse-polyclonal anti-GFP Sigma Aldrich 11814460001 - 1:1000<br>Rabbit-monoclonal anti-pH3 Sigma Aldrich 06570 (clone H3S10P) - 1:1000<br>Goat anti-Mouse IgG (H+L) Secondary Antibody, HRP conjugate Life Technologies 32430 - 1:1000<br>Goat anti-Mouse IgG (H+L) Cross-Adsorbed Secondary Antibody, HRP conjugate, Alexa Fluor 488 Invitrogen A-11001 - 1:1000<br>Goat anti-Rabbit IgG (H+L) Cross-Adsorbed Secondary Antibody, Alexa Fluor Plus 647 Invitrogen A32733- 1:1000
Validation	Mouse monoclonal anti-V5 (clone SV5-pk1) BioRad MCA1630 (PMIDs: 1713260, 7504859, 34038716), • Mouse monoclonal anti-alpha-tubulin (clone 12G10) Developmental Studies Hybridoma Bank AB1157911 (PMID: 7781905), • Mouse-polyclonal anti-GFP Sigma Aldrich 11814460001 (PMID: 24746148), Rabbit-polyclonal anti-pH3 Sigma Aldrich 06570 (PMIDs: 12386157, 18043729)

Eukaryotic cell lines

Policy information about cell lines

Cell line source(s)	HEK293T (ATCC Cat# CRL-1573; RRID:CVCL 0045), Wild-type mouse embryonic fibroblast (MEF) - gift from John Couchman, University of Copenhagen, Denmark. PMID: 20154082, Syndecan-4 knockout MEF - gift from John Couchman, University of Copenhagen, Denmark. PMID: 20154082.
Authentication	None of the cell lines were authenticated.
Mycoplasma contamination	Cell lines were not tested for mycoplasma contamination but no indication of contamination was observed.
Commonly misidentified lines (See ICLAC register)	No commonly misidentified cell lines were used.

Animals and other organisms

Policy information about studies involving animals; ARRIVE guidelines recommended for reporting animal research

Laboratory animals	The following C. elegans strains were used in this study at larval stage 4 and young adult hermaphrodites: AH205, VC602, JK5933, RB690, RJP3846, RJP3867, RJP3866, RJP3857, RJP3855, RJP3863, RJP3858, RJP3746, RJP3747, RJP3845, RJP4118, RJP4729, RJP4730, RJP4744, RJP4035, RJP4731, RJP5186, RJP5187, RJP5188, KHK667, RJP5189, RJP5190, JK4472
Wild animals	No wild animals were used in the study.
Field-collected samples	No field collected samples were used in the study.
Ethics oversight	Ethical approval is not required for Caenorhabditis elegans studies

Note that full information on the approval of the study protocol must also be provided in the manuscript.
